# Thermochemical-dependent Cu(i) siting and mobility in Cu-CHA revealed by XAS and quantum mechanical simulations

**DOI:** 10.1039/d6sc05389k

**Published:** 2026-08-25

**Authors:** Gabriele Deplano, Andrea Martini, Elisa Borfecchia, Janis Timoshenko, Beatriz Roldán Cuenya, Matteo Signorile, Silvia Bordiga

**Affiliations:** a Department of Chemistry, NIS and INSTM Reference Centre, Università di Torino Via P. Giuria 7, 10125 and Via G. Quarello 15/A 10135 Torino Italy matteo.signorile@unito.it; b Department of Interface Science, Fritz-Haber Institute of the Max Planck Society 14195 Berlin Germany

## Abstract

Cu mobility in Cu-zeolites is still an underrated factor in the description of their reactivity, in spite of multiple literature reports suggesting its relevance. Herein we provide a XAS-based description of the displacement of Cu(i) in chabazite upon exposure to different gas-phase ligands and/or thermal treatments. Two preferential sites are accessible to ions depending on the applied stimuli, namely thermodynamically favored in 6-membered rings and metastable in 8-membered rings. DFT-aided data interpretation fully supports the experimental findings, allowing an atomistic description of Cu(i) mobility under the explored conditions.

## Abstract 2

Cu(i) mobility in chabazite is tracked by combining XAS and DFT.

## Graphical abstract

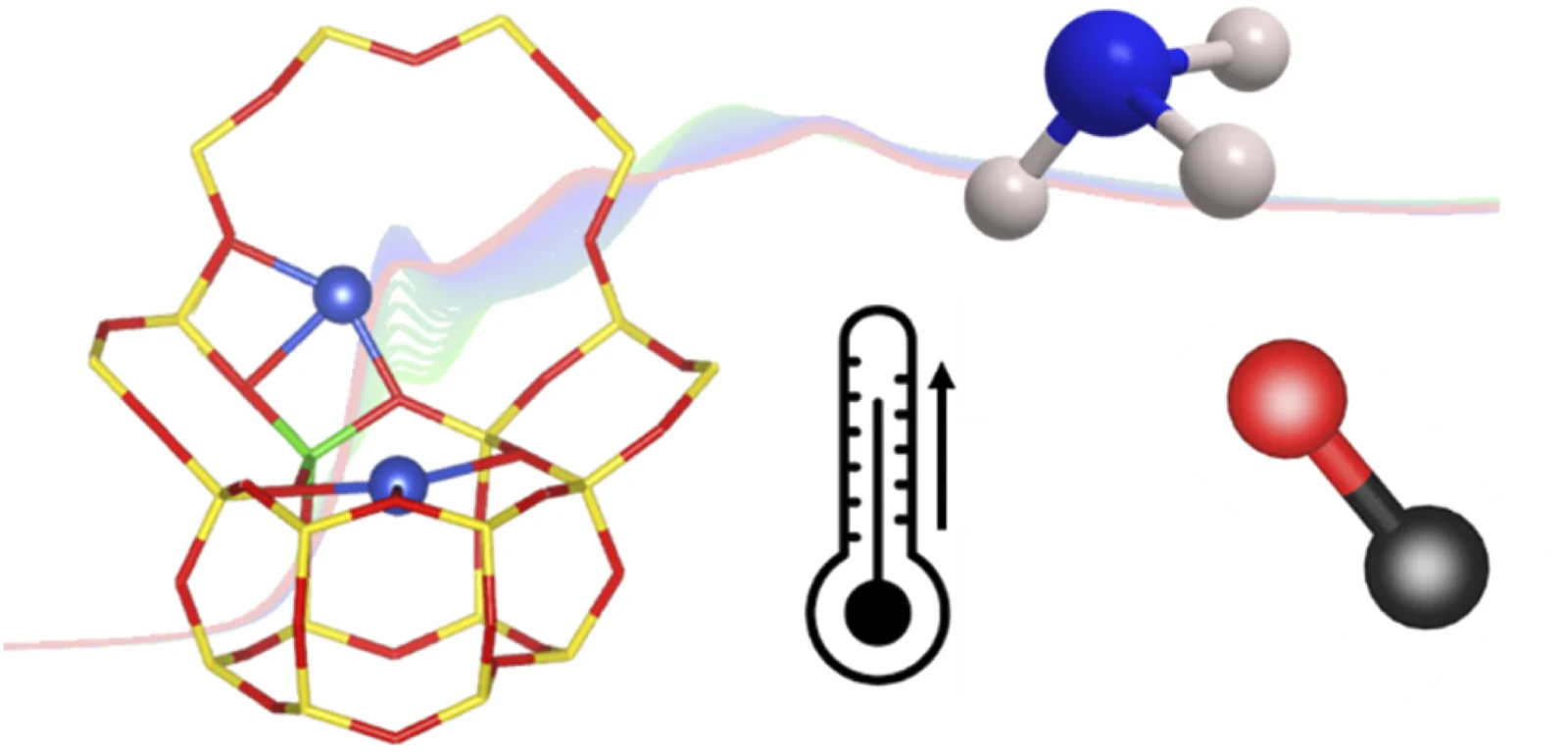

Copper zeolites are well known for their redox catalytic properties, making them well suited for different oxidation/reduction reactions.^1^ Currently, their main application is ammonia-assisted selective catalytic reduction of NO_*x*_ (NH_3_-SCR),^2^ which is essential in the automotive industry for exhaust treatment in diesel engines. Furthermore, Cu-zeolites emerged in the last 20 years as highly selective partial oxidation catalysts, capable of activating strong C–H bonds to yield oxygenated products. A representative process here is the methane to methanol reaction,^3^ where Cu-zeolites ensure selectivity close to 100% in both stepwise and continuous operation modes. The key mechanistic steps in these processes rely on the Cu(i)/Cu(ii) redox couple, with a continuous exchange of oxidation states driving the redox reactivity. An interesting feature of Cu ions with different oxidation states is their distinct behavior in terms of mobility within the aluminosilicate zeolitic framework. Several reports suggested that the mobility of Cu(i) is much higher than that of Cu(ii),^4–6^ in particular in the presence of specific ligands. The most striking case is that of the ammonia-solvated [Cu(i)(NH_3_)_2_]^+^ cations, which can be displaced by approximately 1 nm from the Al site whose negative charge they balance. This mobility has been identified as the main factor for producing key reactive intermediates in NH_3_-SCR. Furthermore, the position of Cu ions with respect to the host zeolite framework has been shown to have an impact on the catalytic performance, in particular, on methane partial oxidation,^7^ by easing the formation of Cu(ii)_*x*_O_*y*_ species that require a specific Cu–Cu distance to be efficiently formed.^8–12^ Accordingly, in addition to knowing the average (equilibrium) placement of Cu ions within the zeolite lattice, the understanding of their mobility under dynamic reaction conditions is important for a comprehensive description of the Cu(i)/Cu(ii) redox chemistry in zeolites. As anticipated, the extent of these displacements falls within the (sub)nanometric range and, unfortunately, any space-resolved experiment would hardly be able to track them. A suitable (though indirect) alternative is provided by spectroscopic methods that can reveal the subtle change in the local environment of Cu ions as a consequence of their displacement within the framework. We investigate herein Cu(i) mobility by X-ray absorption spectroscopy (XAS) in a pre-reduced Cu-chabazite sample (Si/Al = 5, Cu/Al = 0.35, labelled (0.35)Cu-CHA(5) hereafter), that was further exposed to CO to displace Cu(i) ions from their equilibrium positions. A thorough analysis of Cu K-edge XAS data was performed by combining Multivariate Curve Resolution – Alternate Least Squares (MCR-ALS) method with spectral simulation *via* first-principles methods.

In our previous contribution, we optimized a reduction protocol for Cu-zeolites based on the exposure to NH_3_ at 500 °C. Upon outgassing of chemisorbed NH_3_ by inert gas flow and cooling the catalyst to near room temperature, the Cu population was dominated by framework-bound “naked” Cu(i) ions, with negligible contributions from residual Cu(ii). By applying this procedure to (0.35)Cu-CHA(5) (see Fig. 1a), the XAS spectrum of the treated material closely resembled that previously reported by us for thermally reduced Cu-CHA.^13–15^ After consequent interaction with CO, Cu(i) was quantitatively converted to framework-bound [Cu(i)(CO)_2_]^+^ and [Cu(i)(CO)]^+^ species, as previously reported.^13^ After another 30 min in a pure He stream, practically only the [Cu(i)(CO)]^+^ species remained present in the material, as pinpointed by a feature at *ca.* 8981 eV overlapping with the typical 1s → 4p signature of Cu(i) at 8983 eV, and a shoulder on the rising edge at *ca.* 8987 eV.^13,16^ The assignment was consistent with calculations, which showed good agreement with the experiment and assigned the rising-edge features to 
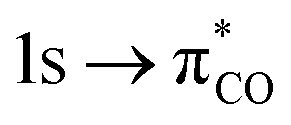
 and 
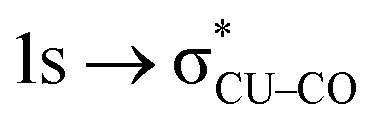
 transitions (Fig. S1). The simulated spectra suggest a preference toward 8MR hosting for [Cu(i)(CO)]^+^ (on the basis of finite difference simulations), though the coexistence of the complex in the 6MR cannot be completely ruled out because of similarities in the spectroscopic fingerprints.

**Fig. 1:**
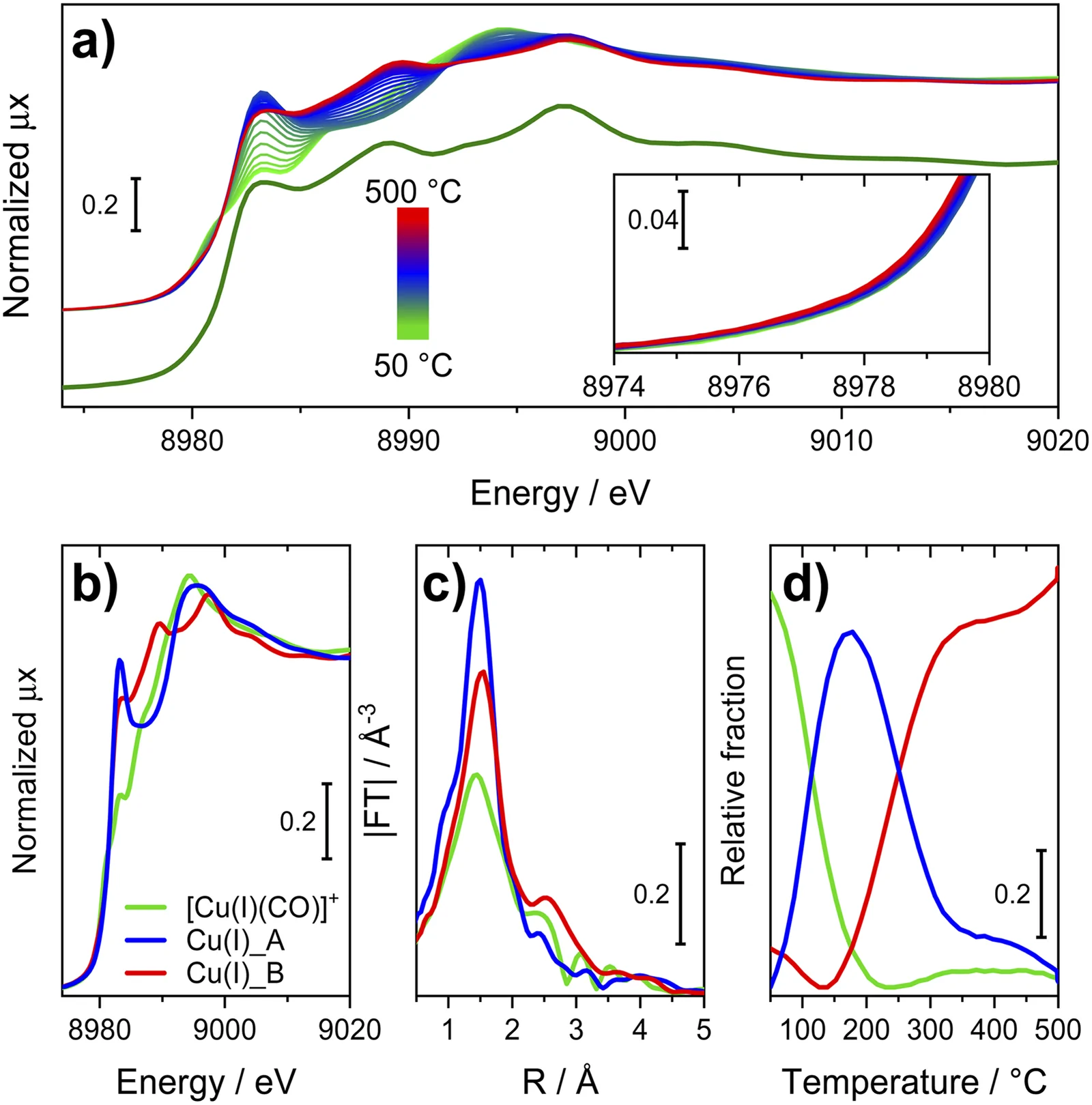
a) *In situ* Cu K-edge XANES spectra of the (0.35)Cu-CHA(5) sample collected during the CO TPD experiment under He flow. The spectrum of (0.35)Cu-CHA(5) after prereduction and prior contact with CO is reported in dark green and vertically shifted for clarity. (b) XANES spectra; (c) moduli of the Fourier-transform (FT)-EXAFS (phase uncorrected) for pure species, as obtained from the TPD dataset by MCR-ALS analysis. (d) Relative concentrations of pure species obtained from the TPD dataset by MCR-ALS analysis.

The *in situ* spectra acquired on (0.35)Cu-CHA(5) during a CO temperature programmed desorption (TPD) protocol are shown in Fig. 1a. While the temperature is increased from 50 °C to 500 °C under He flow, the Cu K-edge XAS spectra undergo substantial modifications.

As the temperature is increased, different species with univocal spectral signatures are observed. The formation of metallic Cu clusters can be excluded here based on XANES and EXAFS wavelet analysis (*vide infra*). Since the most plausible decomposition pathway for [Cu(i)(CO)]^+^ is CO desorption with the restoration of bare Cu(i), the observed species likely correspond to framework-bound Cu(i) ions with different coordination geometries, rather than to species characterized by a different oxidation state (as the absence of the typical Cu(ii) 1s → 3d transition suggests). Since the CHA framework is quite rigid and a rearrangement of its ring system is unlikely, this change in the coordination environment can be ascribed to the relocation of Cu(i) ions to a different binding site. According to previous studies in the literature, Cu ions in CHA preferentially anchor either in the 8- or 6-membered rings (8MR and 6MR, respectively) typical of this zeolite topology.^17^ The site associated with the 8MR is usually characterized by a more T-shaped coordination geometry, whereas Cu(i) hosted in the 6MR site preferentially approaches a trigonal-planar arrangement.

In order to understand these temperature dependent transformations in a quantitative way, an MCR-ALS analysis of the *in situ* spectral profiles during TPD was performed, revealing that 3 species exist and convert into each other as temperature increases. This was based on the scree-plot of PCA and tests with ±1 components with respect to the suggested value. The XANES region of the obtained pure spectra (Fig. 1b), which is particularly sensitive to the coordination geometry, bond angles, and local symmetry, shows clear differences between the initial state of the catalyst (unambiguously assigned to [Cu(i)(CO)]^+^)^13^ and the states of the catalyst observed upon CO desorption and heating, labelled Cu(i)_A and Cu(i)_B hereafter. The Cu(i)_A component, dominating at lower temperature (maximum concentration at *ca.* 180 °C, according to MCR-ALS-yielded concentration profiles shown in Fig. 1d), can be provisionally associated with Cu(i) located in the 8MR site. In particular, the intense absorption feature at *ca.* 8983 eV, assigned to the Cu(i) 1s → 4p transition, is typically enhanced in the case of low-coordinated and quasi-linear Cu(i) environments.^18^ Such intensity enhancement arises from increased 3d–4p orbital mixing, which strengthens the dipole-allowed transition. Conversely, the component prevailing at high temperature (Cu(i)_B, whose concentration becomes dominant above 300 °C) shows a weaker 1s → 4p feature and a more structured rising-edge with an additional, broad peak at *ca.* 8989 eV. This is in agreement with reported spectra of non-linear Cu(i) geometries and therefore possibly ascribable to Cu(i) residing in the 6MR site.^15,17^ Additional differences are observed in the XANES post-edge region between 9000 and 9020 eV; however, the interpretation of features in this energy range is less straightforward, because it is dominated by transitions to highly delocalized states and by multiple-scattering interference between the outgoing photoelectron and neighboring atoms, still strongly affected by the coordination geometry. Interestingly, the Cu(i)_B XANES spectrum is closely similar to that measured on reduced (0.35)Cu-CHA(5) prior to exposure to CO, suggesting that the associated Cu(i) structure is thermodynamically favored upon heating to high temperature, regardless of the previous thermochemical history of the sample.

Additionally, for XANES, MCR-ALS allowed the retrieval of pure contributions of the EXAFS portion of the spectrum for each of the three species. The magnitude of FT-EXAFS, as reported in Fig. 1c, further confirms that the initial [Cu(i)(CO)]^+^ species are well distinguished from Cu(i)_A and Cu(i)_B. Compared to other components, the first shell FT-EXAFS peak of [Cu(i)(CO)]^+^ is strongly dampened. We note that this difference cannot be simply assigned to the difference in thermal disorder, since the first pure species is mostly observed at lower temperatures. Instead, the lower intensity and increased widths of the main FT-EXAFS feature for the first species could indicate the heterogeneity of bond lengths in the monocarbonyl adduct (1C, 2O coordination, 1.8–2.2 Å) compared to that of the Cu attached to the zeolite framework (2O or 3O coordination, 2.0–2.1 Å). The difference among the FT-EXAFS profiles of Cu(i)_A and Cu(i)_B is, instead, less marked: both states show an intense first-shell peak at *R* = 1.50 Å as their main feature, with a lower intensity in the case of the Cu(i)_B component. In this case, the effect of temperature on the peak intensity cannot be ruled out, since a Cu(i)_B species with lower FT-EXAFS peak intensity is observed at significantly higher temperatures with respect to Cu(i)_A. As a result, the lower FT-EXAFS peak intensity for Cu(i)_B does not contradict our initial structural hypothesis derived from XANES analysis that Cu(i)_B has a higher first shell coordination number. Interestingly, even considering this temperature-induced signal dampening, Cu(i)_B presents a more intense and structured second shell peak (*R* = 2.5 Å), that further supports the initial assignment to 6MR-hosted Cu(i). As a matter of fact, in the confined 6MR, Cu(i) is surrounded by more heavy scatterers (Al and Si) in the 3–4 Å range than in a 8MR, which could contribute to the build-up of the second shell peak. To clarify the nature of this second-shell feature, the wavelet transform (WT) of the EXAFS signals was analyzed (Fig. S2). Cu(i)_A WT-EXAFS displays a single lobe centered at *k* ∼4 Å^−1^, characteristic of low-Z scatterers. The Cu(i)_B one shows the same first-shell feature together with an additional ridge at *R* ∼2.5–3.5 Å and *k* ∼ 4–6 Å^−1^, with a maximum near *k* ∼5 Å^−1^. These *k*-values are incompatible with Cu–Cu backscattering (usually appearing at significantly higher *k* values at around 7 Å^−1^; see Fig. S3)^8,19–22^ and instead most likely correspond to scattering from framework atoms (Al/Si), in line with previous propositions.

On the basis of these preliminary spectroscopic considerations, structural models of Cu(i) located in the 8MR and 6MR sites of the CHA framework were constructed and used for theoretical XAS simulations. To establish a quantitative correlation between the spectral features and local structure, theoretical spectra were calculated by time-dependent (TD-)DFT approach within the ORCA code using cluster models extracted from periodic optimized structures. Although this approach can in principle approximate only a portion of an ordered solid, the element-selective and local character of XAS, together with the dilute and weakly interacting nature of Cu in the zeolite framework, justifies this approximation. Indeed, rapid spectral convergence is obtained upon increasing the cluster size (Fig. S4 and S5). Additionally, because orbital-based quantum-chemical approaches do not adequately reproduce the white-line and post-edge region of the XAS spectra, a full multiple-scattering, finite difference approach was employed to simulate the higher-energy part of the edge through the FDMNES code. The combination of these two methods allows us then to reproduce the entire XANES profile and enables a direct correlation between experimental spectra and structural models. Fig. 2 compares the simulated spectra of Cu(i) in the 8MR and 6MR sites, respectively, with the pure spectral components Cu(i)_A and Cu(i)_B extracted by MCR-ALS analysis from the experimental data.

**Fig. 2:**
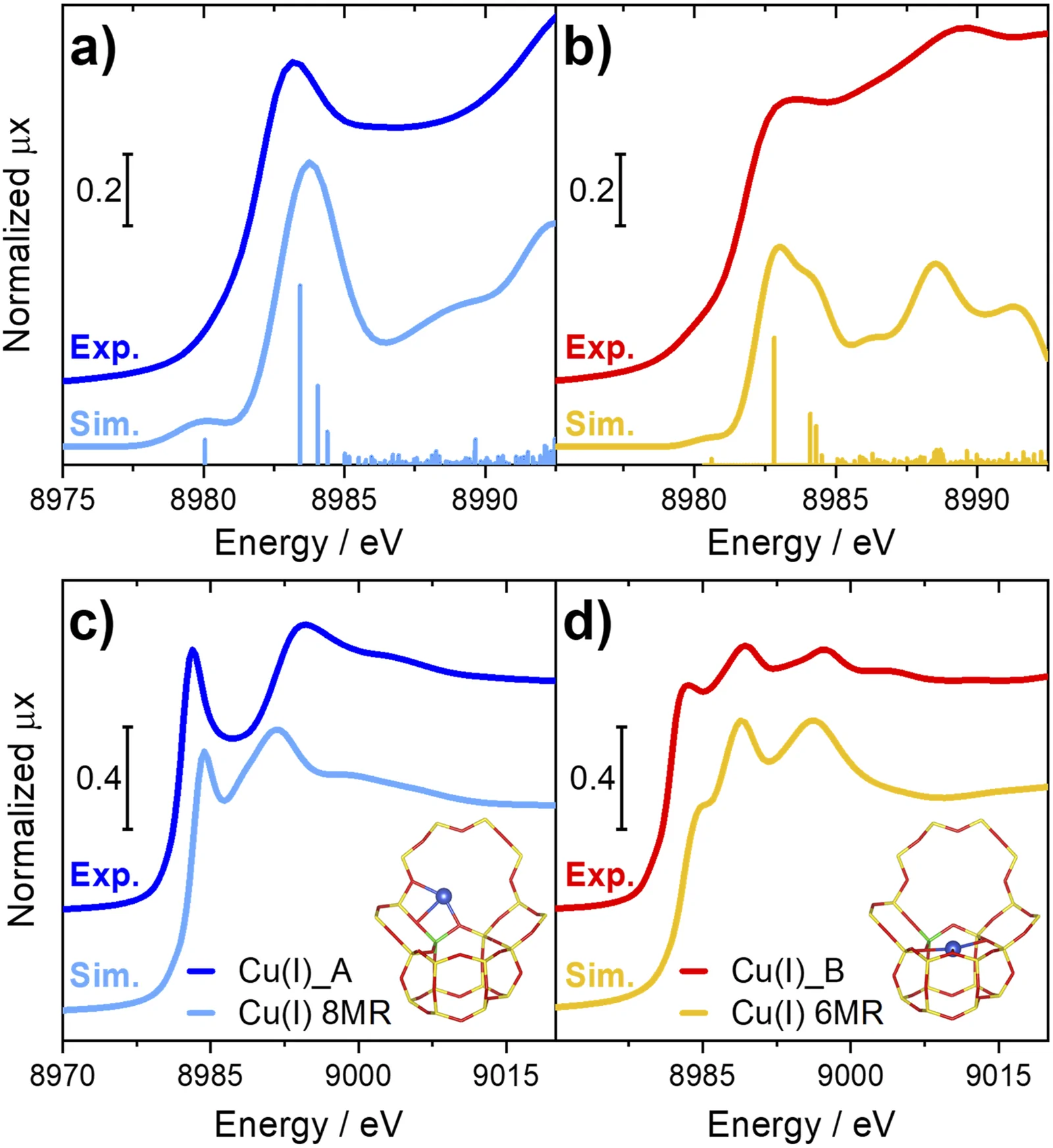
Comparison of the MCR-ALS-yielded Cu K-edge XANES spectra of Cu(i)_A and Cu(i)_B with (a) and (b) TD-DFT simulations for Cu(i) in 8MR and 6MR sites, respectively; and (c) and (d) finite difference simulations for Cu in 8MR and 6MR sites, respectively. Vertical bars in panels (a) and (b) show the energy and intensity (prior convolution with Gaussian peak functions) of the main electronic transitions from TD-DFT calculations. Insets of panels (c) and (d) depict structural models of the Cu sites in the 6MR and 8MR (Cu, blue; Si, yellow; Al, green; O, red).

TD-DFT calculations (Fig. 2a and b) could capture the main differences between the two species, in particular concerning the rising-edge region, by predicting a sharper and more intense 1s → 4p transition for the T-shaped coordination motif of Cu(i) in the 8MR, that nicely matches the spectrum of the Cu(i)_A component. Concerning the Cu(i)_B species, the 1s → 4p transition is less intense and broader than for the Cu(i)_A component, a feature that is consistently captured by the TD-DFT simulated spectrum of Cu(i) in the 6MR environment. By analyzing the output of the TD-DFT calculations, and in agreement with previously established descriptors in the literature,^23,24^ this trend is correlated with a stronger p–d mixing in the case of Cu in the 8MR (Fig. S6). This stronger mixing arises from the larger O–Cu–O bond angle for the Cu in the 8MR site, in comparison to that for Cu in the 6MR (144° *vs. ca.* 120°, respectively). Furthermore, the 6MR model could reproduce the rising-edge feature at around 8988 eV in the Cu(i)_B spectrum (arising from the transitions to highly delocalized virtual states), while this contribution is dampened for the 8MR model. Thus, based on the rising-edge feature comparison in MCR-derived and TD-DFT simulated spectra, the Cu(i)_A component is likely ascribable to Cu hosted in the 8MR, where it can assume a quasi-linear geometry. On the other hand, a 6MR-hosted Cu site with trigonal coordination is more representative of the Cu(i)_B state. This initial assignment is further confirmed by finite difference simulations performed with the FDMNES code, based on the same structural models used in TD-DFT calculations (Fig. 2c and d). In agreement with the TD-DFT results, the 8MR model produces a simulated spectrum that better resembles Cu(i)_A. Although the agreement in terms of energy for the main post-edge features is not perfect, as is already well known for XANES simulations of Cu-exchanged zeolite systems,^25^ the relative intensities and the overall shape of the simulated spectrum resemble those in the experimental data. An even better agreement is obtained among the simulated spectrum for the 6MR model and the experimental Cu(i)_B, with a quantitative reproduction of all rising- and post-edge spectral features.

In order to cross-validate the structural interpretation derived from the computational XANES investigation, we then focused on the quantitative analysis of the EXAFS region of the spectra (additional data provided in Fig. S7), which provides direct structural information in real space about the distribution of nearest neighbors around the Cu center.

A conventional EXAFS analysis based on a restricted set of single- and multiple-scattering paths becomes particularly challenging for Cu sites in CHA, where medium-range framework contributions are significant. In these systems, multiple-scattering signals arise from well-defined Cu–O–(Al/Si) geometries within the small zeolite rings, making the EXAFS signal strongly dependent on bond angles and local topology.^15,17^ As a consequence, the parametrization of scattering paths requires explicitly considering such multiple-scattering paths and/or simplifying the fit by neglecting terms, imposing constraints, *etc.* In addition, the Cu coordination environment is intrinsically heterogeneous, leading to non-Gaussian bond-length distributions that cannot be reliably described using a small number of Debye–Waller factors. This produces strong correlations among the fitting parameters and limits the structural uniqueness of conventional least-squares refinements.

To overcome these limitations, we therefore adopted a Reverse Monte Carlo (RMC) fitting approach to fit the obtained EXAFS data.^26,27^ The DFT-derived models for the 8MR and 6MR geometries were used as starting points for RMC-EXAFS simulations to fit the Cu(i)_A and Cu(i)_B EXAFS spectra, respectively, by using the RMC procedure implemented in the EvAX code. Herein, the DFT-derived structural models were further adjusted by allowing constrained random displacements of the atoms around their initial coordinates, with the goal to minimize the difference between the experimental and simulated EXAFS spectra. Thereby, RMC simulations account for the static and thermal disorder while preserving the overall topology and coordination environment. This procedure naturally includes in the fit contributions from the distant coordination shells and multiple-scattering contributions without explicit path parametrization. RMC-EXAFS refinement provides an independent structural validation of our structure models established based on XANES analysis and DFT simulations. Fig. 3a and b compares the experimental FT-EXAFS with the best-fit curves obtained from the RMC simulations for the Cu(i)_A and Cu(i)_B components. The agreement between experiments and simulations is excellent, with a *R*_factor_ of 4% for Cu(i)_A and 6% Cu(i)_B (computed as defined in ref. 28), demonstrating that the structural models accurately reproduce the experimental EXAFS signal as well. The corresponding radial distribution functions (RDFs) extracted from the final RMC configurations are reported in Fig. 3c and d. For the Cu(i)_A species, the RDF displays a sharp and intense maximum centered at ∼1.96 Å, corresponding to the Cu–O bond in the first coordination shell. The peak is narrow and nearly symmetric, indicating a well-defined and weakly disordered local environment around the Cu center. This feature arises from two oxygen atoms directly coordinated to Cu and is consistent with the structural model placing the Cu(i) site in the 8-membered ring of the CHA framework in a quasi-linear O–Cu–O geometry. At larger radial distances (2–4 Å), the RDF becomes significantly broader, reflecting a wide distribution of Cu-framework distances involving framework O, Al and Si atoms. Such a broad distribution indicates that these more distant scattering paths are characterized by a large spread in interatomic distances and angles. As a consequence, their EXAFS contributions are strongly dampened, which explains the absence of well-resolved higher-*R* features in the FT magnitude. In contrast, for the Cu(i)_B species, the RDF shows a first-shell maximum at approximately the same position in *R*-space as observed for Cu(i)_A and assigned again to Cu–O scattering. Compared to the Cu(i)_A case, this peak is slightly broader, consistent with an increase in oxygen coordination around the Cu center and reflecting the transition from the quasi-linear twofold coordination to a higher number of nearest neighbors. At larger radial distances, the RDF reveals more localized contributions associated with framework atoms (O, Al and Si) around 3.0–3.5 Å, which give rise to the second shell peak observed in the FT-EXAFS at *ca.* 2.5 Å (phase uncorrected). The appearance of this feature indicates that the distribution of Cu-framework distances is narrower for Cu(i)_B species than in the Cu(i)_A case, resulting in more pronounced FT-EXAFS contributions from the zeolite lattice within the rigid and narrow 6MR.

**Fig. 3:**
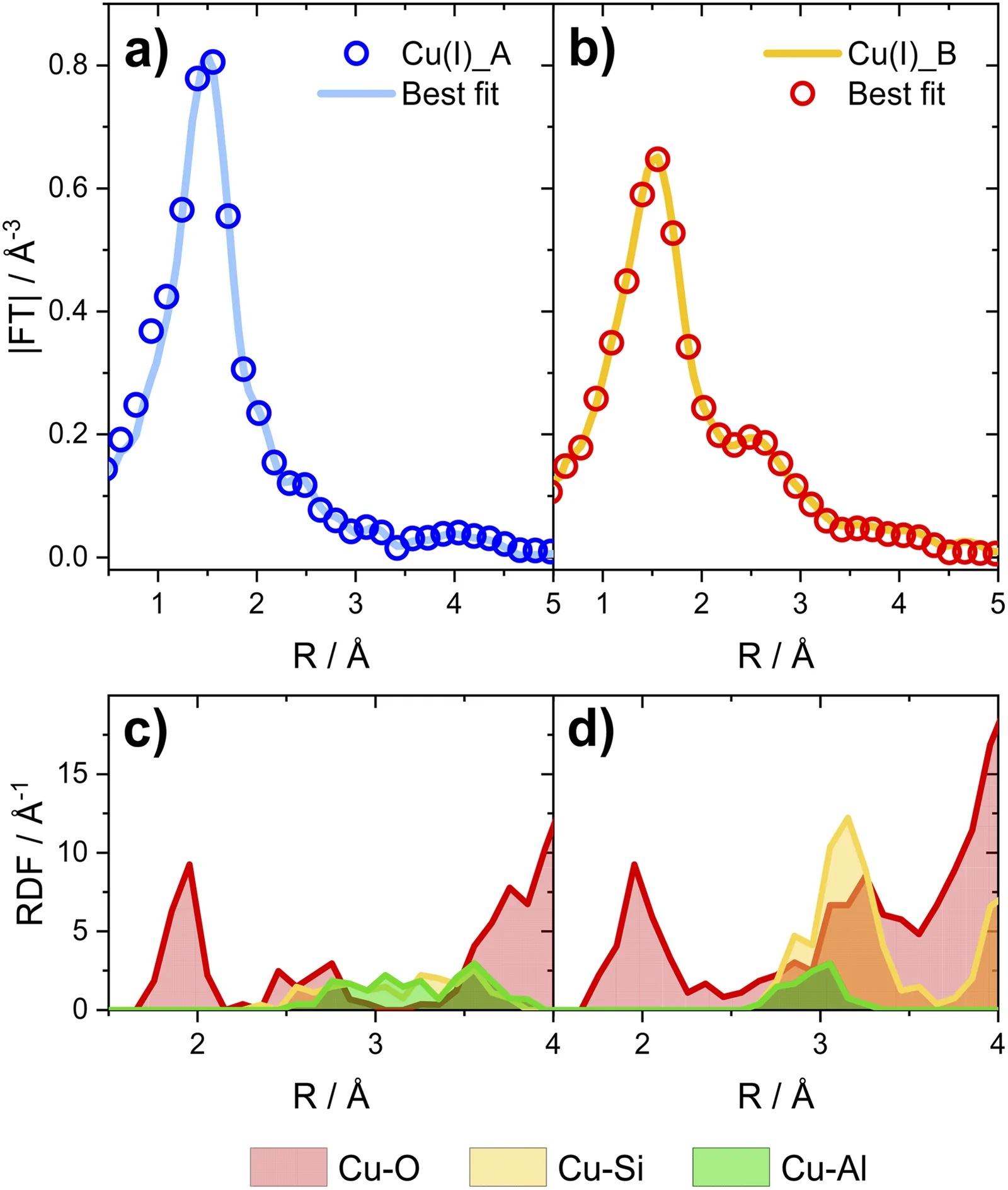
Comparison of the experimental FT-EXAFS for (a) Cu(i)_A; and (b) Cu(i)_B species with the corresponding best Reverse Monte Carlo (RMC) fits (phase uncorrected). One point each of three is shown in the MCR-ALS pure component spectra for the sake of readability. Radial distribution functions (RDFs, phase corrected) around the Cu absorber extracted from the final RMC structure model, showing the partial contributions of Cu–O, Cu–Si and Cu–Al paths for (c) Cu(i)_A; and (d) Cu(i)_B.

The combination of all gathered information allows us to propose a consistent scheme to describe the Cu(i) mobility within the CHA framework as a response to precise thermochemical stimuli, as presented in Fig. 4a.

**Fig. 4:**
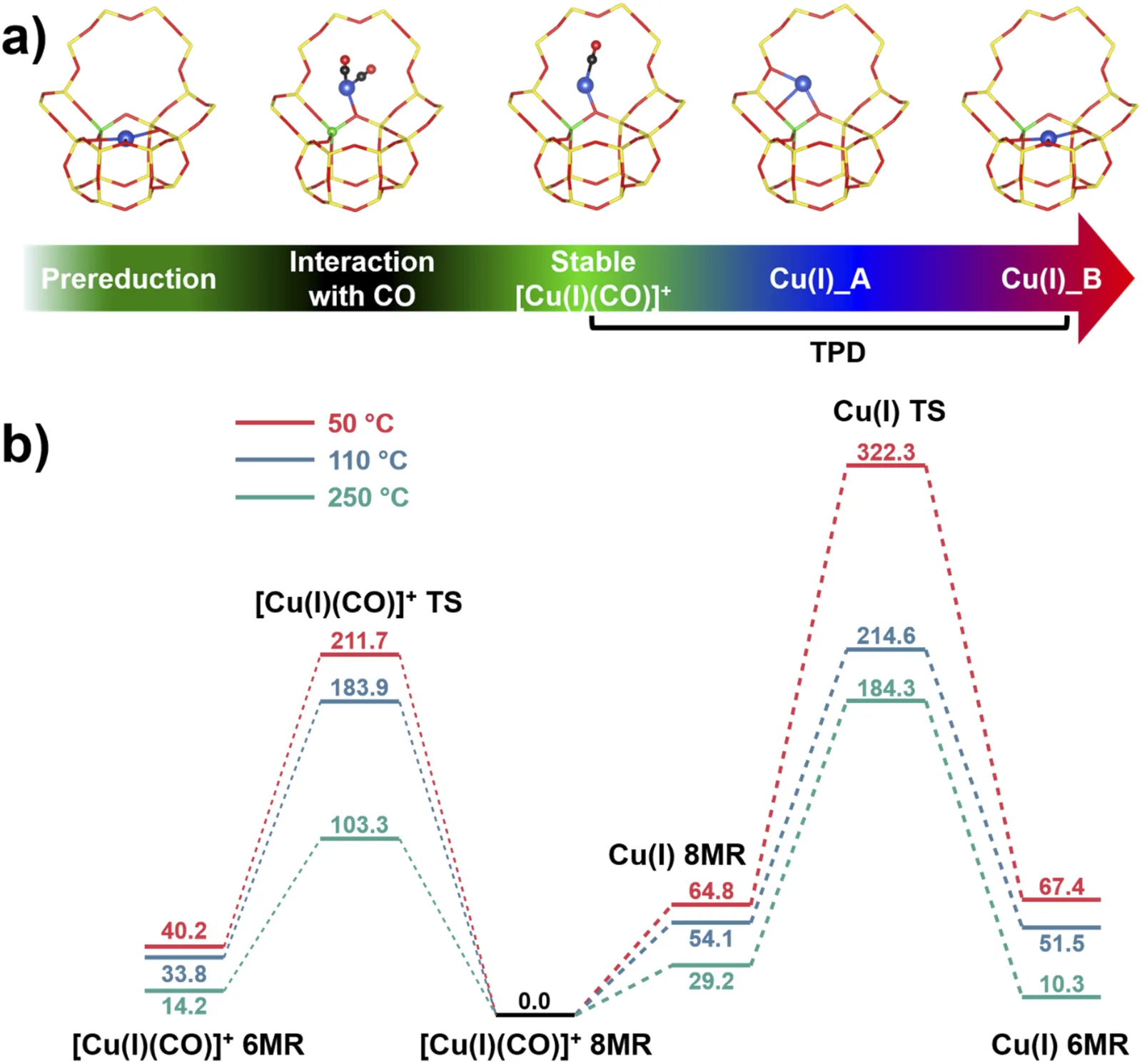
a) Scheme of proposed Cu(i) thermochemical mobilization in pre-reduced Cu-CHA undergoing CO exposure and its subsequent temperature programmed desorption, based on the quantitative analysis of XANES and EXAFS results. (b) DFT potential energy surface for processes potentially involved during the TPD stage. Gibbs free energy values are reported in kJ mol^−1^. Structural models are shown in Fig. S8.

Upon the initial prereduction procedure, the larger fraction of Cu(i) is hosted in the 6MR, as suggested by the close similarity of the XANES spectrum collected prior to the exposure to CO to the MCR-derived spectrum for Cu(i)_B species. As the sample is exposed to CO, as previously reported,^13^ Cu(i) ions are converted to framework-bound [Cu(i)(CO)_2_]^+^ complexes: by interaction with CO, Cu(i) is then displaced from the 6MR to the 8MR. By desorbing excess CO with a pure He stream at 50 °C, the [Cu(i)(CO)_2_]^+^ complexes are rapidly and quantitatively converted to the [Cu(i)(CO)]^+^ monocarbonyl form. Afterwhile, during the TPD stage, the decomposition of [Cu(i)(CO)]^+^ initially leads to the formation of the Cu(i)_A species, assigned to a bare Cu(i) ion in quasi-linear coordination within the 8MR. Cu(i) ions can then back-migrate to their thermodynamically favored 6MR site (represented by Cu(i)_B) when a sufficient thermal input is provided. As a matter of fact, Cu(i)_B becomes the dominant component in terms of concentration only at *ca.* 250 °C, inferring the existence of an energy barrier to Cu(i) diffusion between the two ring systems.

This spectroscopy-based TPD mechanism is quantitatively supported by the results of DFT calculations, as shown in Fig. 4b. Since a variable temperature range is involved, Δ*G* values have been computed at 50 °C (representative of the starting point of the process), 110 °C (when the concentration of Cu(i)_A overcomes that of [Cu(i)(CO)]^+^) and 250 °C (as Cu(i)_B becomes more abundant than Cu(i)_A). A fixed CO partial pressure was considered along the whole process, considering it an upper bound derived from maximal CO release as estimated from the MCR concentration profile of [Cu(i)(CO)]^+^. As the TPD begins at 50 °C, [Cu(i)(CO)]^+^ sits in the 8MR (taken as the energy reference), as supported by the unfavorable energetics associated with [Cu(i)(CO)]^+^ in the 6MR (+40.2 kJ mol^−1^). Under this condition, CO is strongly bound to Cu(i), and required to overcome a 64.8 kJ mol^−1^ barrier to achieve desorption. As temperature increases, the desorption barrier decreases, making CO release more likely in line with expectations. The naked Cu(i) ions, positioned in the 8MR, then require a an energy input as large as 160.5 kJ mol^−1^ at 110 °C to overcome the transition state (labeled TS Cu(i) in Fig. 4b) allowing their diffusion toward the 6MR site. The barrier progressively decreases as temperature increases (155.1 kJ mol^−1^ at 250 °C), supporting the progressive migration of Cu(i) from 8MR to thermodynamically stable 6MR sites observed in the high temperature regime. As an additional cross-check, an alternative path involving the diffusion of [Cu(i)(CO)]^+^ from the 8MR to the 6MR followed by desorption has been considered, even though unlikely on an experimental basis. The DFT results show that the barrier imposed by the transition state for [Cu(i)(CO)]^+^ diffusion (referred to as TS [Cu(i)(CO)]^+^) is much higher than the energy penalty for CO desorption in the low temperature regime (where [Cu(i)(CO)]^+^ concentration is maximal), amounting to 211.7 kJ mol^−1^ at 50 °C (*vs.* 64.8 kJ mol^−1^ required for desorption). Accordingly, this side path has a very low probability of occurrence, further supporting the likeliness of the main proposed mechanism.

Overall, this work demonstrates the potential of combining XAS, first-principles simulations and RMC-EXAFS fitting to track the subnanometric diffusion of Cu(i) ions within the CHA framework, paving the way for future studies linking Cu mobility to catalytic activity in Cu-zeolites.

## Materials and methods

Synthesis and basic characterization of the (0.35)Cu-CHA(5) sample studied in this work is detailed in previous publications.^29^*In situ* XAS measurements were performed in transmission mode at the BM23 beamline^30^ of the European Synchrotron Radiation Facility (ESRF), under the beamtime awarded through the CH-6264 proposal.^31^ Samples were prepared as self-supporting pellets of optimized thickness and the thermochemical protocol was performed within a Microtomo reactor cell, developed by the ESRF sample environment group.

Multivariate curve resolution (MCR) using the alternating least squares (ALS) algorithm was applied to XAS spectra using the graphical user interface developed by Jaumot *et al.* in the MATLAB environment.^32^

Reverse Monte Carlo (RMC) EXAFS fits were performed using the EvAX code.^26,28^ The initial structural models were constructed from the DFT-optimized geometries of Cu(i) located in the 8MR and 6MR sites of the CHA framework.

Structural models for Cu(i) species were obtained within a DFT framework using the CRYSTAL17 periodic code^33^ and the hybrid B3LYP functional with a DZVP quality basis set, as already described in detail in a previous study.^13^

TD-DFT calculation of pre-edge and rising-edge XANES spectra was performed using the ORCA software (version 5.0.3),^34–36^ on cluster models cut from the optimized periodic structures. The TD-DFT calculations were performed within the Tamm–Dancoff approximation using the range-separated hybrid GGA CAM-B3LYP functional^37^ with the ZORA approximation^38^ to account for relativistic effects, in conjunction with the ZORA-def2-TZVP(-f) basis set.^39^

Finite-difference calculation of rising- and post-edge XANES spectra was performed using the finite difference method as implemented in the FDMNES code^40,41^ with a cluster radius of 6 Å; convolution parameters were selected with Cu_2_O as the reference, as reported in Table S1, with the aid of the PyFitit software.^42^

Extended experimental and computational details are provided in the SI.

## Author contributions

G. D.: conceptualization, methodology, formal analysis, investigation, writing – original draft, visualization. A. M.: methodology, formal analysis, writing – original draft, visualization. E. B.: supervision. J. T.: writing – review & editing. B. R. C.: writing – review & editing. M. S.: methodology, investigation, writing – original draft, visualization, data curation, supervision. S. B.: supervision, project administration, funding acquisition.

## Conflicts of interest

There are no conflicts to declare.

## Supplementary Material

SC-017-D6SC05389K-s001

## Data Availability

Data for this article, including raw data for all figures, are available at Edmond (the Open Research Data Repository of the Max Planck Society) at https://doi.org/10.17617/3.ZTBKRF. Supplementary information (SI): extended materials and methods; NTO analysis of TD-DFT simulated spectra of [Cu(i)(CO)]^+^ in 8MR and 6MR; wavelet transform analysis of EXAFS signal of Cu(i)_A and Cu(i)_B; convergence test for simulated XANES spectra *vs.* model size; NTO analysis of TD-DFT simulated spectra of Cu(i) in 8MR and 6MR; additional EXAFS data; DFT structures. See DOI: https://doi.org/10.1039/d6sc05389k.
