# Supplementary material for: Thermochemical-dependent Cu(i) siting and mobility in Cu-CHA revealed by XAS and quantum mechanical simulations
Source: Chem Sci. 2026 Aug 25;17(41):19830–7. doi: 10.1039/d6sc05389k (PMC13539381; doi:10.1039/d6sc05389k)
Supplement: Supplementary file 1 [file SC-017-D6SC05389K-s001.pdf]

Supplementary Information (SI) for Chemical Science.
This journal is © The Royal Society of Chemistry 2026

Supplementary Information for

Thermochemical-dependent Cu(I) siting and mobility in Cu-CHA revealed by XAS and quantum mechanical simulations

Gabriele Deplano[1,†], Andrea Martini[2], Elisa Borfecchia[1], Janis Timoshenko[2], Beatriz Roldan Cuenya[2], Matteo Signorile[1*] and Silvia Bordiga[1]

[1] Department of Chemistry, NIS and INSTM Reference Centre, Università di Torino, Via P. Giuria 7, 10125 and Via G. Quarello 15/A, 10135, Torino, Italy

[2] Department of Interface Science, Fritz-Haber Institute of the Max Planck Society, 14195 Berlin, Germany

[†] Present address: Department of Inorganic Spectroscopy, Max Planck Institute for Chemical Energy Conversion, Stiftstraße 34–36, 45470 Mülheim an der Ruhr, Germany

* Corresponding author: Matteo Signorile, matteo.signorile@unito.it

S1. Extended materials and methods

The synthesis and characterization of the (0.35)Cu-CHA(5) sample studied in this work is detailed in previous publications.[1] The composition of the sample was determined by ICP and resulted in a Si/Al = 5 and a Cu/Al = 0.35.

In situ XAS measurements were performed at the BM23 beamline of the European Synchrotron Radiation Facility (ESRF), under the beamtime awarded through the CH-6264 proposal.[2] The Microtomo reactor cell,[3] developed by the ESRF sample environment group, was chosen as sample environment. The powdered Cu-zeolites were prepared in the form of self-supporting pellets with thickness optimized for transmission measurements and placed inside the reactor cell. The temperature of the sample was controlled by the heating system integrated in the Microtomo cell. The total gas flow rate and the composition of the feed were adjusted by a set of mass flow controllers controlled by a remote terminal. Treatment of the sample consisted of a pre-reduction step in NH_3 (Air Products, 0.4 mol% in He) at 500 °C, followed by flushing in pure He (Air Products) at the same temperature; after cooling and stabilization at 50 °C under He flow, CO (Air Liquide, 5 mol% in He) was dosed on the reduced samples. The gas flow

was then switched to pure He, and a temperature-programmed desorption (TPD) step up to 500 °C (heating rate 5 °C/min).
The Cu K-edge spectra were acquired in transmission mode, employing a double-crystal Si(111) monochromator to scan the incident energy, and ionization chambers to detect incident (I_0) and transmitted photons (I_1). A Cu metal foil was measured simultaneously with all the collected XAS spectra by means of a third ionization chamber (I_2), for energy calibration purposes.[4,5] The chambers were filled with a He/Ar mixture up to 2bar with Ar partial pressure of 0.1 and 0.3 bar for I_0 and $I_{1,2}$ chambers, respectively. A full XAS acquisition (8830–9800 eV energy range) required ca. 2 min/scan. Spectra were collected in continuous mode with an energy step of 0.3 eV, then were rebinned with constant energy steps of 5 eV in the pre-edge region (8928 – 8960 eV) and 0.3 eV in the XANES region (8960 – 9050 eV); a uniform sampling step in k-space of 0.08 $Å^{-1}$ was adopted in the EXAFS region. A custom Python-based script based on the Larch library was employed for data alignment and normalization.
Multivariate curve resolution (MCR) using the alternating least squares (ALS) algorithm was applied to the XAS spectra using the graphical user interface developed by Jaumot et *al.* in the MATLAB environment. The number of relevant PCs (n) was selected by locating the elbow point of the relative Scree plot, visually inspecting the residual variances and evaluating the goodness of the fit in the interval k-1≤ n ≤ k+1. The convergence tolerance on the Lack of Fit (LOF) parameter for two successive iterations was set to 0.1%. After selection of the PCs, the MCR-ALS routine was initialized using the SIMPLISMA method. Non-negativity was imposed on both spectral and concentration profiles, as well as closure (i.e. sum to 1) for concentrations. The convergence was achieved in less than 25 iterations, with final LOF values <0.5%.

Wavelet transform (WT) analysis was performed using an in-house Python implementation based on the convolution of the $k^2\chi(k)$ signal with a Morlet mother wavelet.[6,7] The wavelet parameters were set to σ=1, defining the width of the Gaussian envelope, and η=6, corresponding to the dimensionless frequency of the oscillatory component. These values were chosen to improve the discrimination of scattering contributions in the R-k domain and to enhance the visibility of higher-shell scattering features located at radial distances around 3 Å.

Reverse Monte Carlo (RMC) EXAFS refinements were performed using the EvAX code.[8,9] The initial structural models were constructed from the DFT-optimized geometries of Cu(I)

located in the 8MR and 6MR sites of the CHA framework. These models were used as starting configurations for the refinement of the experimental EXAFS spectra. The passive electron reduction factor S_0^2 and the energy shift ΔE were fixed to 0.85 and 0 eV, respectively, for both structural models. These parameters were determined independently by fitting the first coordination shell of a Cu_2O reference compound and were kept constant during the RMC refinement. During the RMC procedure, atoms were allowed to undergo small random displacements around their equilibrium positions in order to account for thermal and static disorder, while preserving the connectivity of the zeolite framework and the coordination environment of the Cu center. The maximum allowed deviation from the initial atomic positions was limited to 0.4 Å for all atoms. For each generated structural configuration, theoretical EXAFS spectra were calculated including both single- and multiple-scattering contributions. Photoelectron scattering amplitudes and phase shifts were obtained using the FEFF8.5L code,[10,11] considering multiple-scattering paths up to eight legs. Structural modifications were accepted or rejected according to the improvement of the fit quality. This process was repeated thousands of times, progressively refining the 3D structure model until a satisfactory agreement between experimental and theoretical EXAFS spectra was obtained. For both conditions, the EXAFS fitting procedure was performed directly in the wavelet transform domain, using a k-range of 2.4-12 $Å^{-1}$ and an R-range of 1-6 Å. The WT analysis employed a Morlet mother wavelet with the same resolution parameters described above. The quality of the fit was assessed using the R-factor criterion defined in ref.[8]

Structural models for the all Cu(I) species were obtained within a density functional theory (DFT) framework using the CRYSTAL17 periodic code[12] employing the B3LYP hybrid functional[13,14] in conjunction with a DZVP quality basis set composed as: the basis set by Nada et al. for describing Si and O atoms;[15] that by Catti et al. for Al;[16] and the Ahlrichs TZVP basis set for Cu, C and O belonging to carbon monoxide.[17] The truncations for the mono- and bi-electronic integral (TOLINTEG) were set to {7 7 7 7 25}. The sampling in the reciprocal space (SHRINK) was set to {3 3} for CHA, providing a total of 14 k points sampled. The maximum order of shell multipoles in the long-range zone for the electron-electron Coulomb interaction (POLEORDR keyword) was chosen to be 6. All the other parameters were set to default values according to the CRYSTAL17 manual.[18] Harmonic frequencies were computed on all structures to ensure they are true minima/transition states. Thermodynamic functions (i.e. Gibbs free energy) were evaluated by applying the canonical partition function.

The calculation of the pre-edge and rising-edge XANES spectra was performed in the frame of time-dependent density functional theory (TD-DFT) using the ORCA software (version 5.0.3).[19–21] Cluster models of the involved species were cut from the optimized periodic structures described previously using the Moldraw software,[22] including the absorber element and its nearest neighbouring atoms, saturating any dangling bond on the Si with H atoms (Si-H bond distance fixed at 1.45 Å). Systematically more complex clusters were cut to include only Cu and the first, second, or third O-Si units shells around Al, respectively; in cases in which small rings would be cut off too crudely with this approach, the whole ring structure was kept instead. Spectra were calculated on these cluster models and showed converged spectral features already at the mid-complexity level (*i.e.*, Cu, adsorbed molecules, two Si-O shells around Al plus saturating H atoms) and were thus used for all TD-DFT calculations. An example of such structures and the convergence of the associated simulated spectral profiles are portrayed in Figure S1 for clarity. The TD-DFT calculations were performed within the Tamm-Dancoff approximation using the range-separated hybrid GGA CAM-B3LYP functional.[23] The ZORA approximation[24] was employed to account for relativistic effects, in conjunction with the ZORA-def2-TZVP(-f) basis set recontracted for the purpose by Pantazis and coworkers.[25] The auxiliary SARC/J basis set[25–28] was used in conjunction with the resolution of identity (RI) approximation for Coulomb integrals (as is default in ORCA 5.0). Spin-orbit coupling was considered using the spin-orbit mean field approximation (SOMF(1X)), and TightSCF settings ($2.5\cdot10^{-7}$ Ha energy tolerance) were used for wavefunction convergence. The excitation space was mapped from the 1s core orbital of Cu to any unoccupied state up to 300 roots, with a maximum value of the Davidson expansion space of 900 (MaxDim 900). Contributions from the electric quadrupole were considered (DoQuad True) and natural transition orbitals (NTO) were calculated to aid in the interpretation of the spectra (DoNTO true); NTOs were rendered using the orca_plot utility and visualized using the VESTA software.[29] All spectra were obtained by applying Gaussian broadening of a 2.3 eV width to the calculated electronic transitions via the orca_mapspc utility and shifted by -5 eV to better match experimental spectra.

Calculation of rising- and post-edge spectra were performed using the FDMNES code[30,31] with a cluster radius of 6 Å and including quadrupole contributions; calibration was performed based on the experimental and simulated spectra of Cu_2O, as reported in Table S1, with the aid of the PyFitit software.[32]

Table S1. Convolution parameters (in eV) for FDMNES-computed XANES spectra, as derived from a reference material (Cu_2O).

Energy_range	*EFermi*	*Ecent*	*Elarg*	*Gamma_hole*	*Gamma_max*	*Shift*
8964-9060	8977	30.7	30.7	1.684	17.69	150

S2. NTO analysis of TD-DFT simulated spectra of $[Cu(I)(CO)]^+$ in 8MR and 6MR

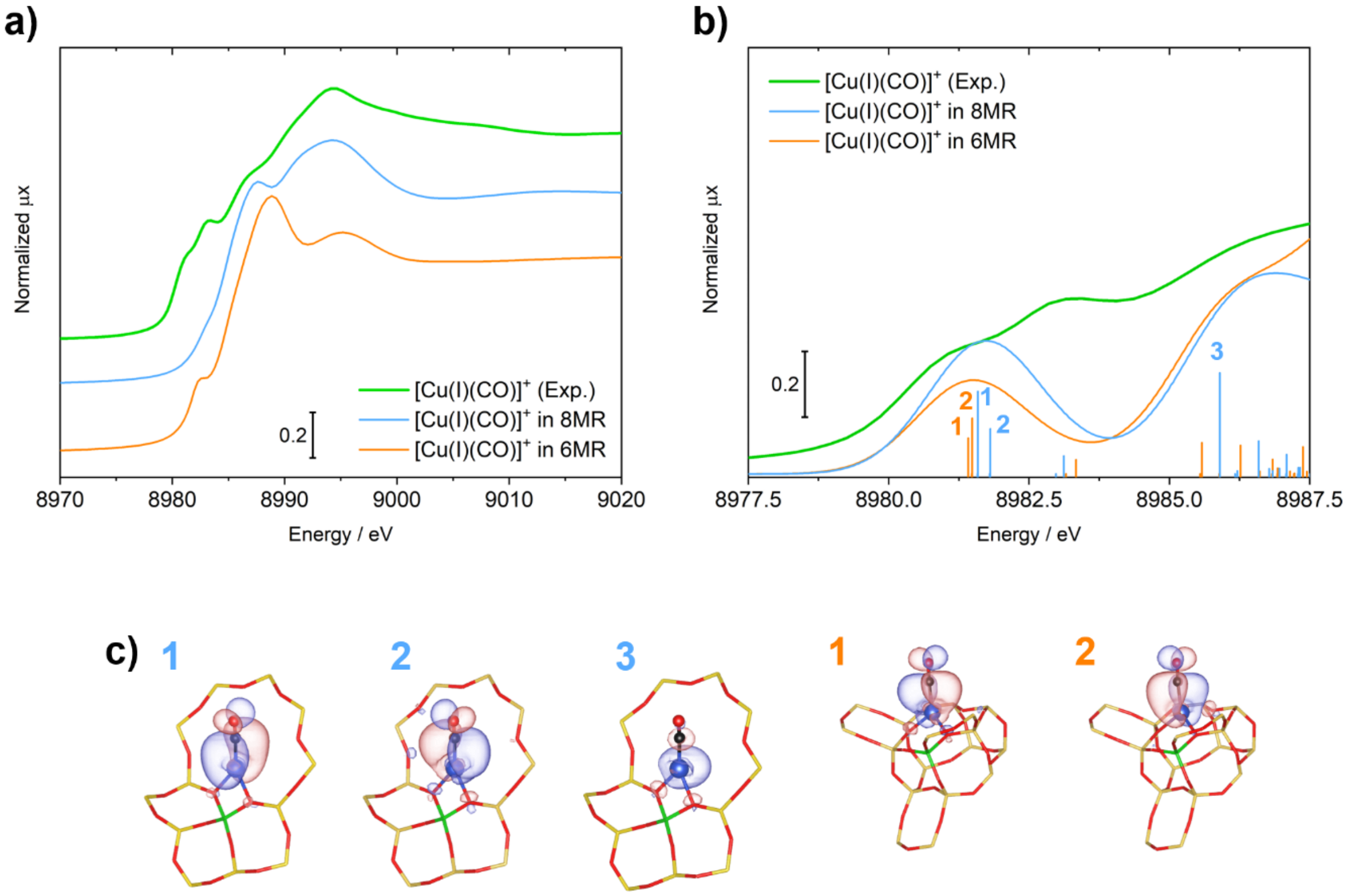

Figure S1. a) Finite difference; and b) TD-DFT simulated spectra for $[Cu(I)(CO)]^+$ in 8MR and 6MR compared to the corresponding experimental spectrum from MCR-ALS. c) Natural Transition Orbitals (NTO) representation of acceptor orbitals for selected transitions, as obtained from TD-DFT calculations (Cu, blue; Si, yellow; Al, green; O, red; C, black).

S3. Wavelet transform analysis of EXAFS signal of Cu(I)_A and Cu(I)_B

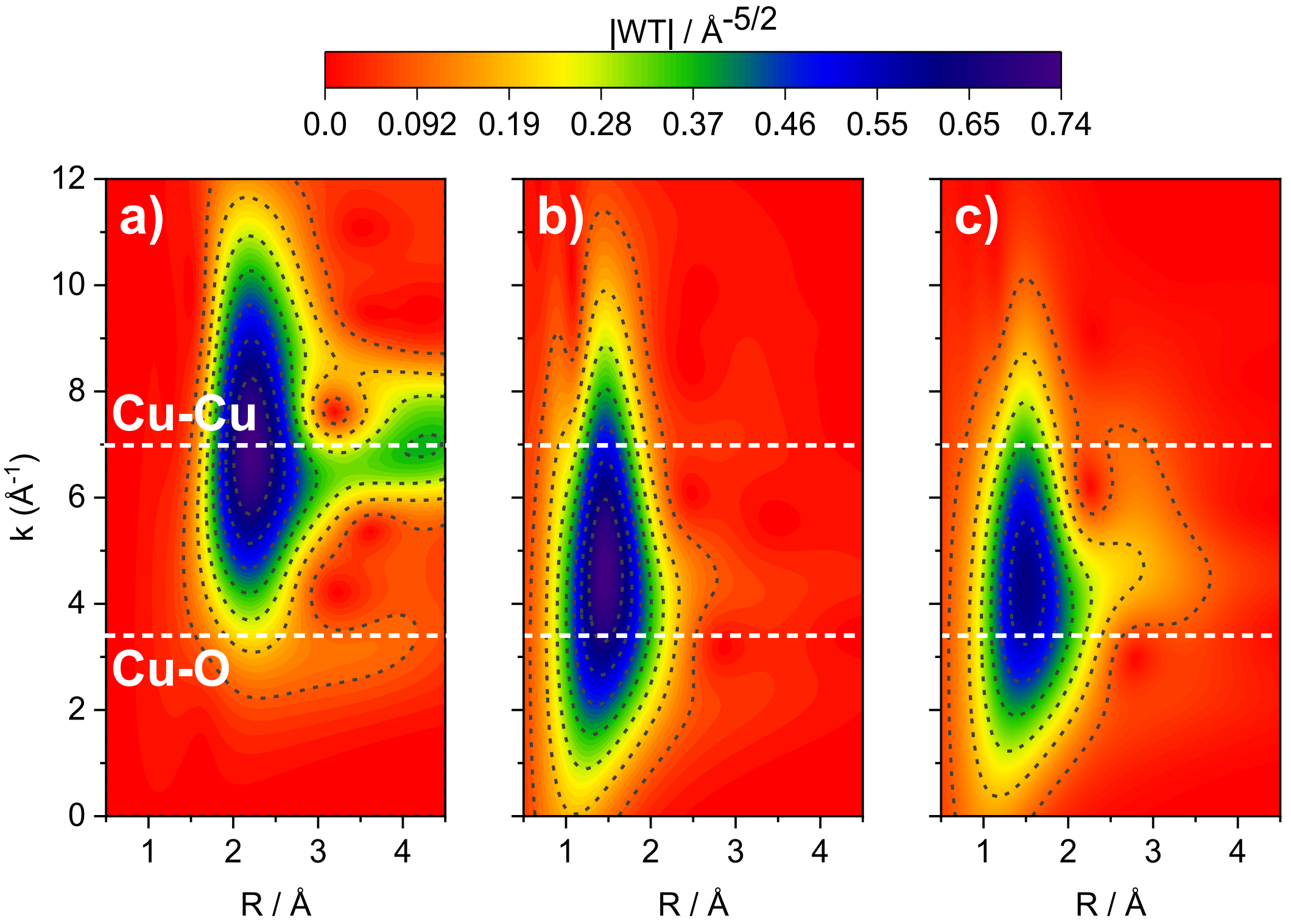

Figure S2. Wavelet transform (WT) modulus maps of the EXAFS signals for: a) Cu metal reference; b) Cu(I)_A; and c) Cu(I)_B.

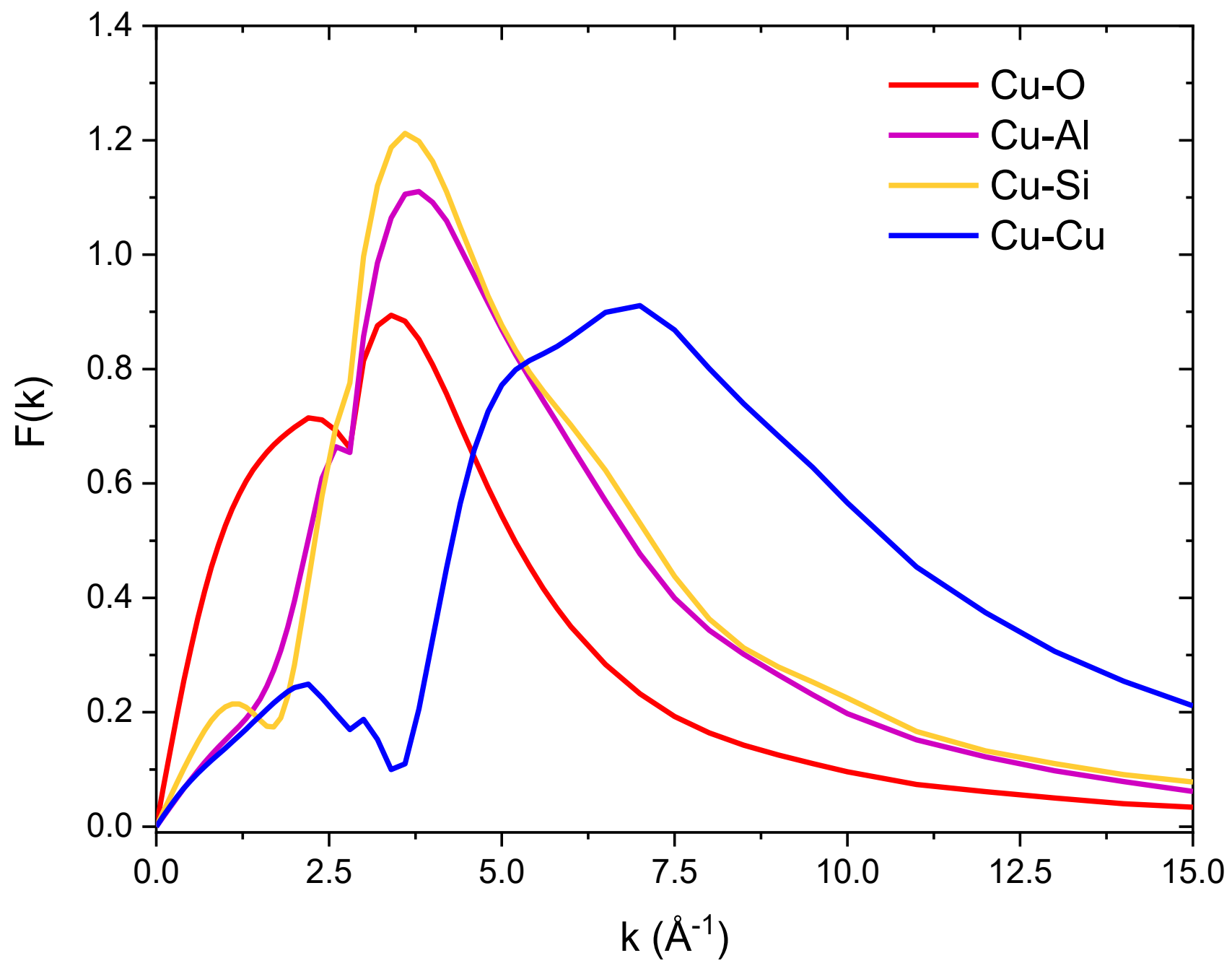

Figure S3. FEFF-derived backscattering amplitude factors F(k) extracted for the Cu-O (red), Cu-Si (yellow), Cu-Al (purple) and Cu-Cu (blue) single scattering contribution.

S4. Convergence test for simulated XANES spectra vs. model size

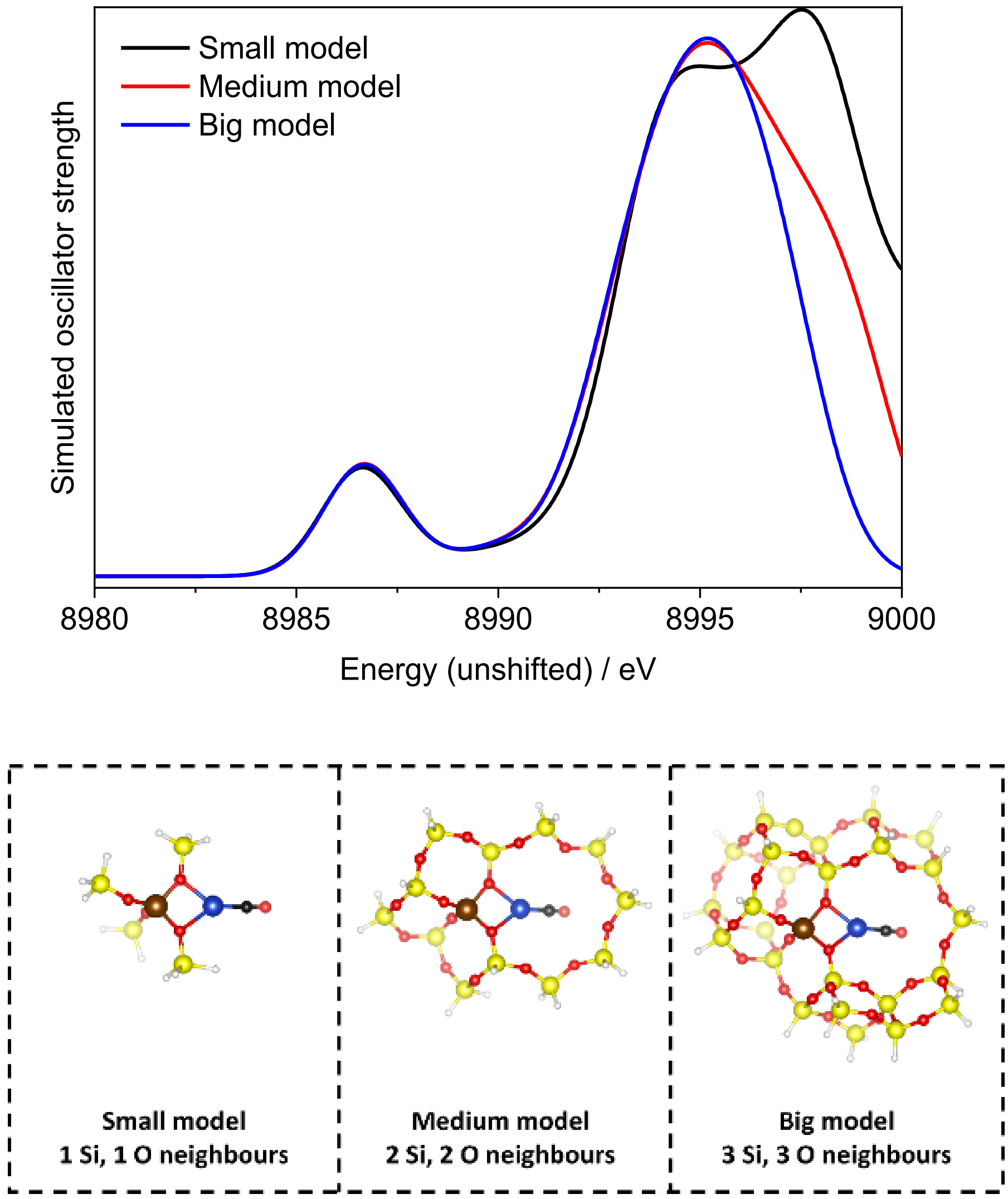

Figure S4. Convergence test for TD-DFT XANES simulations as a function of cluster model size.

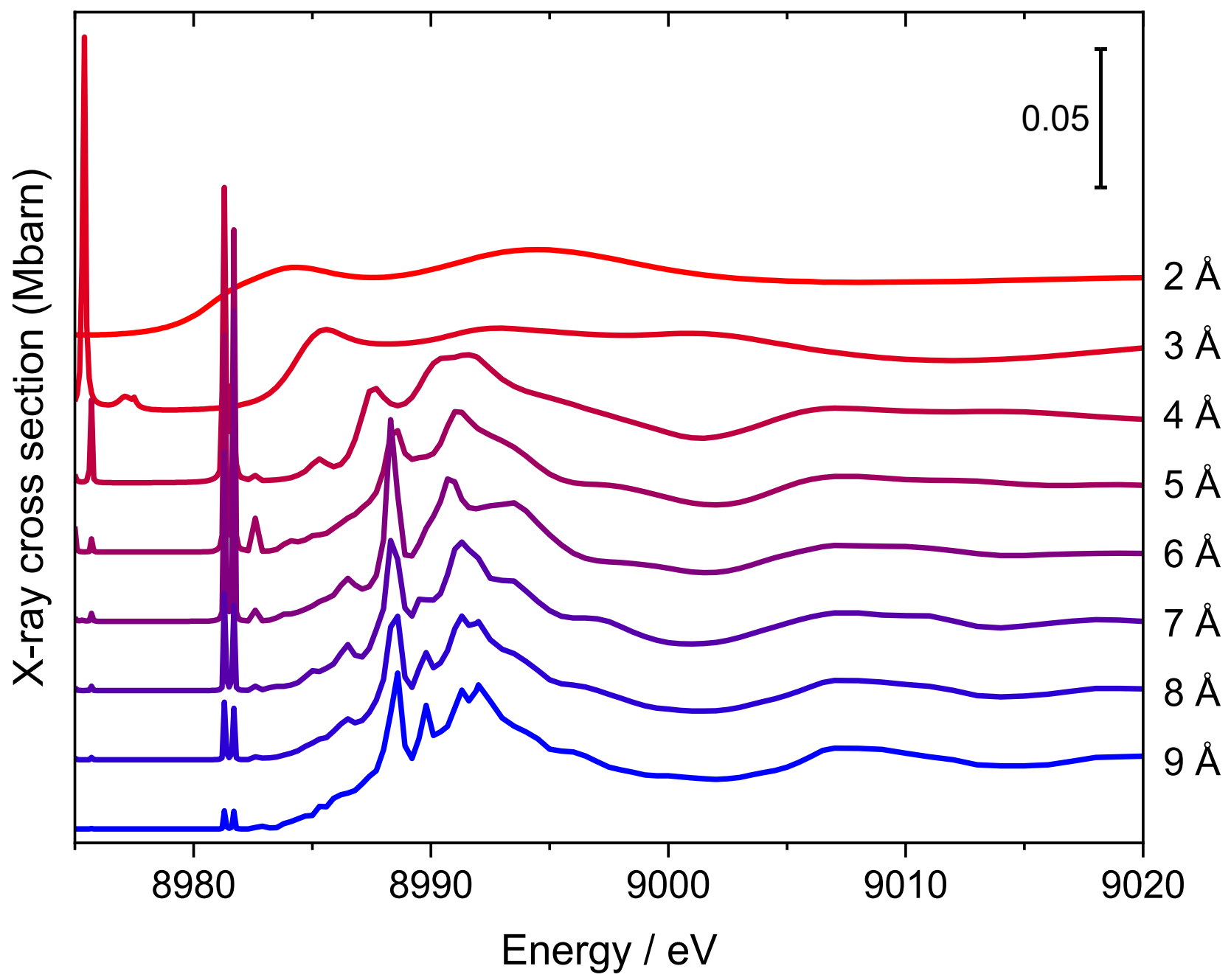

Figure S5. Convergence test for finite-difference XANES simulations as a function of cluster model size. Curves are vertically shifted for the sake of visualization.

S5. NTO analysis of TD-DFT simulated spectra of Cu(I) in 8MR and 6MR

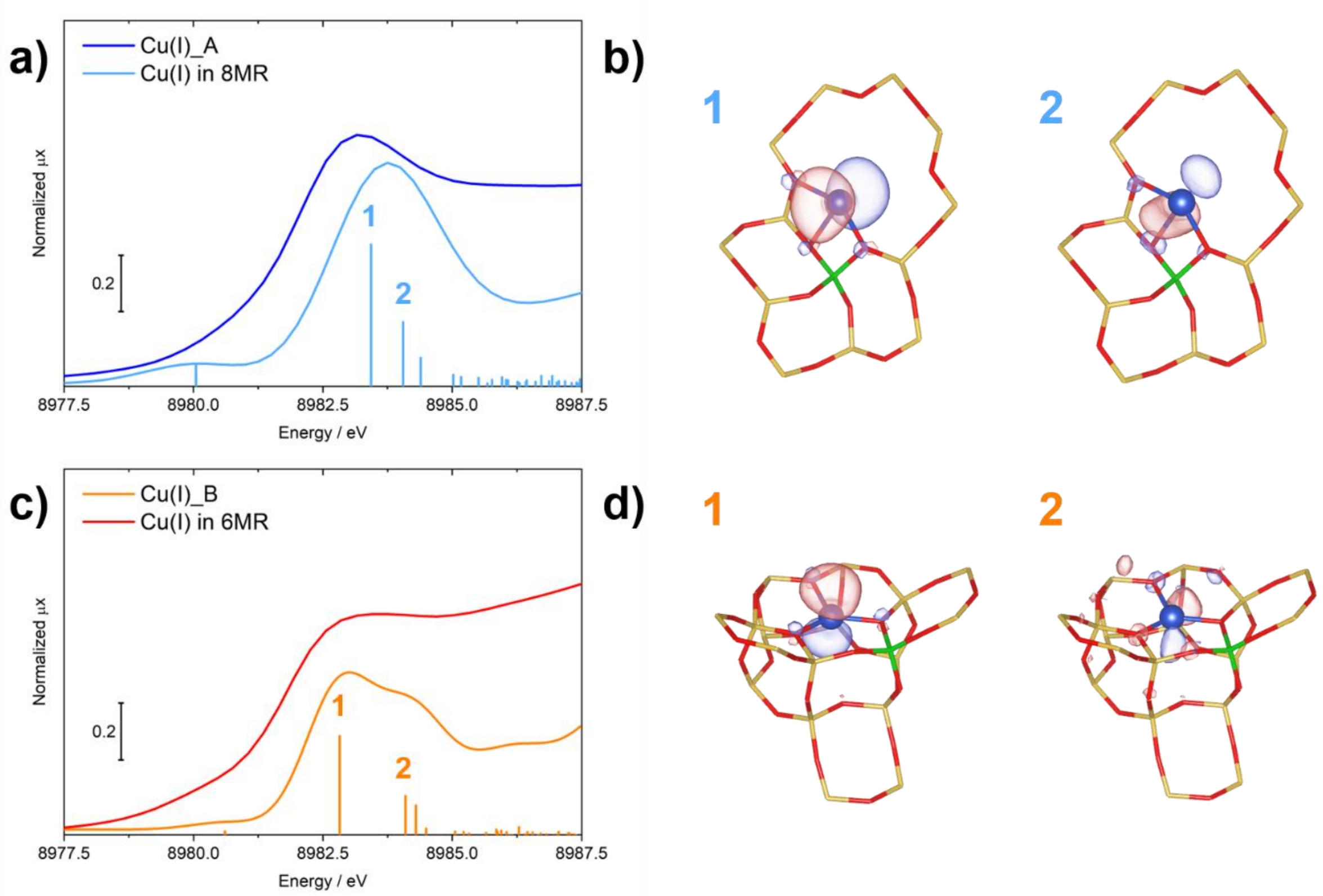

Figure S6. TD-DFT simulated spectra for Cu(I) in a) 8MR; and c) 6MR compared to the corresponding experimental spectrum from MCR-ALS. b,d) Natural Transition Orbitals (NTO) representation of acceptor orbitals for selected transitions (Cu, blue; Si, yellow; Al, green; O, red; C, black).

S5. Additional EXAFS data

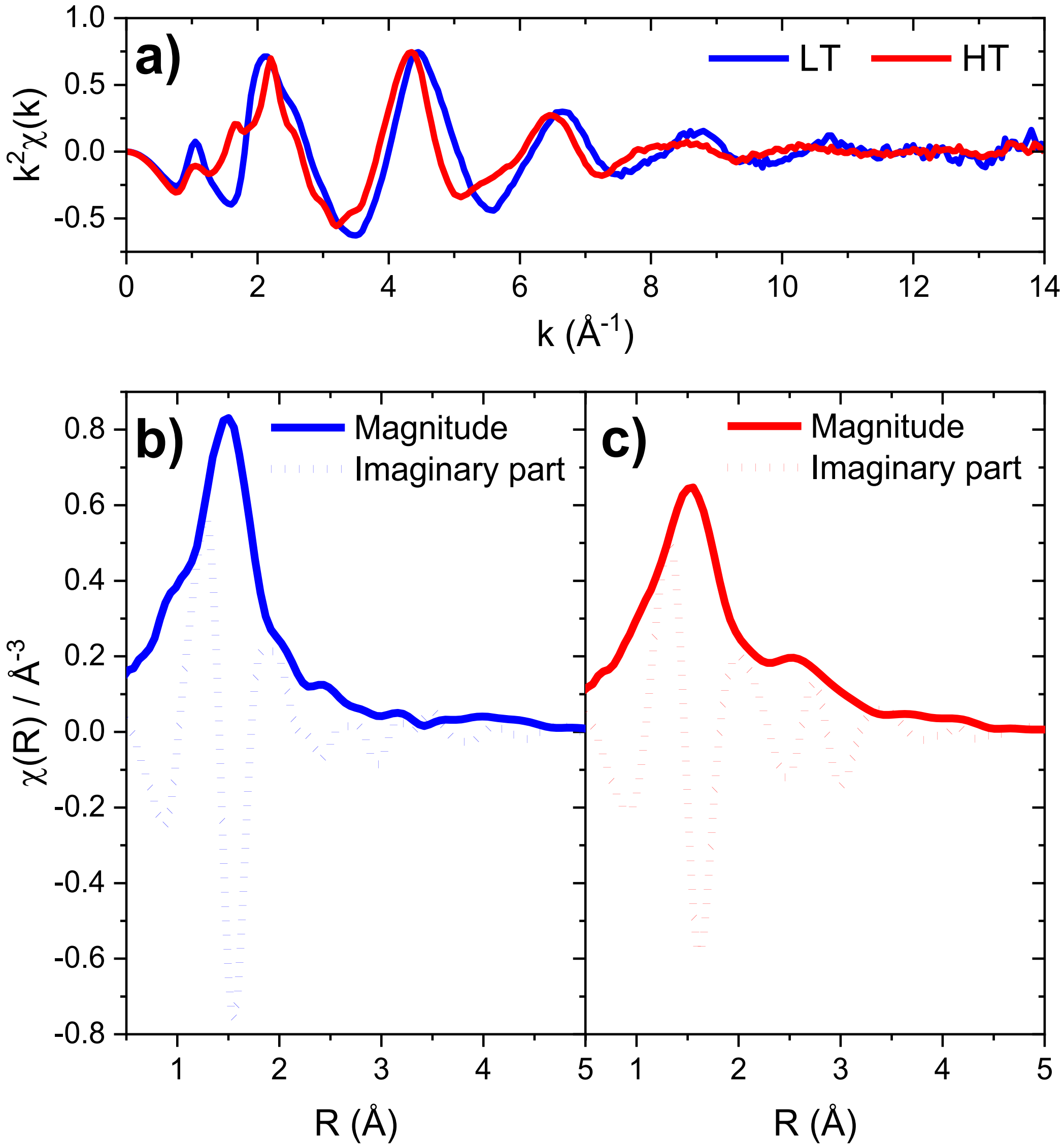

Figure S7. a) Raw $k^2\chi(k)$ EXAFS spectra of LT and HT pure components; phase uncorrected modulus (solid) and imaginary part (dotted) of the experimental FT EXAFS of b) LT; and c) HT pure components.

S6. DFT structures

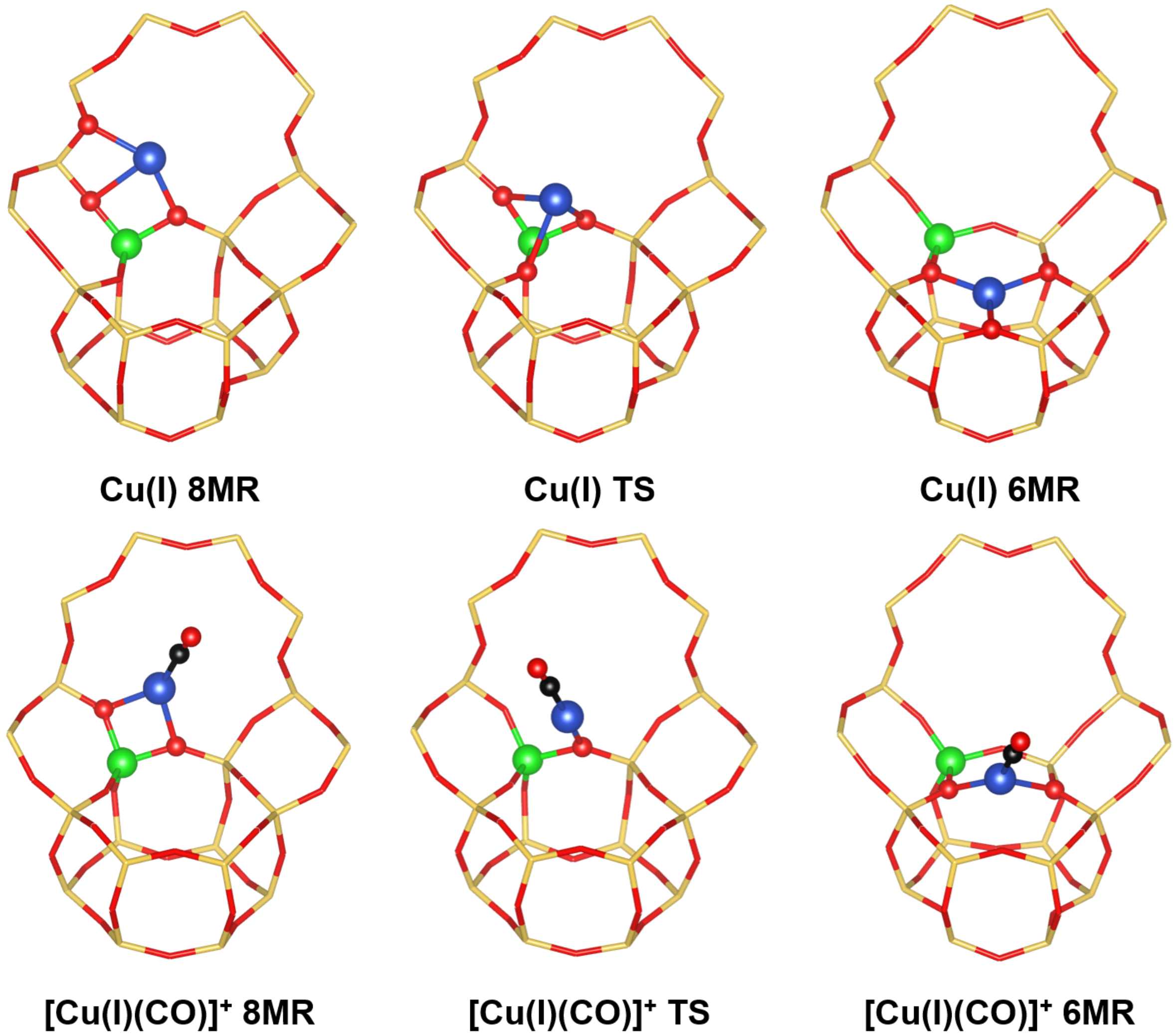

Figure S8. Graphical representation of all DFT optimized structures, including minima and transition states (Cu, blue; Si, yellow; Al, green; O, red; C, black).

The minimum of Cu(I) in 8MR presents a T-shape coordination, with two oxygen in quasi linear coordination at Cu-O distances of 2.036 and 2.157 Å and making a O-Cu-O angle of 143.6°. The third oxygen coordinates Cu orthogonally with Cu-O distance of 2.365 Å.

The minimum of Cu(I) in 6MR has a planar triangular coordination in the plane of the 6MR, with Cu-O distances of 1.993, 2.055 and 2.118 Å.

The transition state separating these two minima shows Cu(I) tri-coordinated on top of Al, with Cu-O distances expanded in comparison with values of minima structures, up to 2.092, 2.353 and 2.437 Å.

The minimum for the $[Cu(I)CO]^+$ adduct in 8MR has a planar triangular coordination involving 2 framework O atoms and C from the CO molecule, with Cu-O distances of 2.049 and 2.105 Å and Cu-C distance of 1.840 Å.

The minimum for the $[Cu(I)CO]^+$ adduct in 6MR also has a planar triangular coordination involving 2 opposite framework O from the 6MR atoms and C from the CO molecule, with Cu out of the plane of the 6MR. The Cu-O distances are 2.019 and 2.195 Å and Cu-C distance is 1.867 Å.

The transition state separating the two $[Cu(I)CO]^+$ minima presents Cu(I) linearly coordinated by a framework O and C from CO, with Cu-O distance of 1.920 Å and Cu-C distance of 1.844 Å.

References

1 T. V. W. Janssens, H. Falsig, L. F. Lundegaard, P. N. R. Vennestrøm, S. B. Rasmussen, P. G. Moses, F. Giordanino, E. Borfecchia, K. A. Lomachenko, C. Lamberti, S. Bordiga, A. Godiksen, S. Mossin and P. Beato, *ACS Catal.*, 2015, **5**, 2832–2845.

2 https://doi.esrf.fr/10.15151/ESRF-ES-780223803.

3 D. Bellet, B. Gorges, A. Dallery, P. Bernard, E. Pereiro and J. Baruchel, *J. Appl. Crystallogr.*, 2003, **36**, 366–367.

4 C. Lamberti, S. Bordiga, F. Bonino, C. Prestipino, G. Berlier, L. Capello, F. D'Acapito, F. X. i Xamena and A. Zecchina, *Phys. Chem. Chem. Phys.*, 2003, **5**, 4502–4509.

5 S. Bordiga, E. Groppo, G. Agostini, J. A. van Bokhoven and C. Lamberti, *Chem. Rev.*, 2013, **113**, 1736–1850.

6 J. Timoshenko and A. Kuzmin, *Comput. Phys. Commun.*, 2009, **180**, 920–925.

7 A. Martini, M. Signorile, C. Negri, K. Kvande, K. A. Lomachenko, S. Svelle, P. Beato, G. Berlier, E. Borfecchia and S. Bordiga, *Physical Chemistry Chemical Physics*, 2020, **22**, 18950–18963.

8 J. Timoshenko, A. Kuzmin and J. Purans, *Comput. Phys. Commun.*, 2012, **183**, 1237–1245.

9 J. Timoshenko, A. Anspoks, A. Kalinko and A. Kuzmin, *physica status solidi (c)*, 2014, **11**, 1472–1475.

10 A. L. Ankudinov, B. Ravel, J. J. Rehr and S. D. Conradson, *Phys. Rev. B*, 1998, **58**, 7565.

11 J. J. Rehr, J. J. Kas, M. P. Prange, A. P. Sorini, Y. Takimoto and F. Vila, *C. R. Phys.*, 2009, **10**, 548–559.

12 A. Erba, J. Baima, I. Bush, R. Orlando and R. Dovesi, *J. Chem. Theory Comput.*, 2017, **13**, 5019–5027.

13 A. D. Becke, *J. Chem. Phys.*, 1993, **98**, 1372–1377.

14 C. Lee, W. Yang and R. G. Parr, *Phys. Rev. B*, 1988, **37**, 785–789.

15 A. C. Nada, R.; Nicholas, J.B.; McCarthy, M.I.; Hess, *Int. J. Quantum Chem.*, 1996, **60**, 809–820.

16 R. D. and M. C. M. Catti, G. Valerio, *Phys. Rev. B*, 1994, **49**, 14179–14187.

17 A. Schäfer, C. Huber and R. Ahlrichs, *J. Chem. Phys.*, 1994, **100**, 5829–5835.

18 R. ; et al. Dovesi, R.; Saunders, V.R.; Roetti, C.; Orlando, .

19 F. Neese, *Wiley Interdiscip. Rev. Comput. Mol. Sci.*, 2012, **2**, 73–78.

20 F. Neese, *Wiley Interdiscip. Rev. Comput. Mol. Sci.*, 2022, **12**, e1606.

21 F. Neese, F. Wennmohs, U. Becker and C. Riplinger, *Journal of Chemical Physics*, 2020, **152**, 2241098.

22 P. Ugliengo, D. Viterbo, G. Chiari, P. Ugliengo, D. Viterbo and G. Chiari, *Z. Kristallogr. Cryst. Mater.*, 1993, **208**, 383–384.

23 T. Yanai, D. P. Tew and N. C. Handy, *Chem. Phys. Lett.*, 2004, **393**, 51–57.

24 E. Van Lenthe, J. G. Snijders and E. J. Baerends, *J. Chem. Phys.*, 1996, **105**, 6505–6516.

25 J. D. Rolfes, F. Neese and D. A. Pantazis, *J. Comput. Chem.*, 2020, **41**, 1842–1849.

26 D. A. Pantazis and F. Neese, *J. Chem. Theory Comput.*, 2009, **5**, 2229–2238.

27 D. A. Pantazis and F. Neese, *Theoretical Chemistry Accounts 2012 131:11*, 2012, **131**, 1292-.

28 D. A. Pantazis, X. Y. Chen, C. R. Landis and F. Neese, *J. Chem. Theory Comput.*, 2008, **4**, 908–919.

29 K. Momma and F. Izumi, *J. Appl. Crystallogr.*, 2008, **41**, 653–658.

30 Y. Joly, *Phys. Rev. B*, 2001, **63**, 125120.

31 S. A. Guda, A. A. Guda, M. A. Soldatov, K. A. Lomachenko, A. L. Bugaev, C. Lamberti, W. Gawelda, C. Bressler, G. Smolentsev, A. V Soldatov and Y. Joly, *J. Chem. Theory Comput.*, 2015, **11**, 4512–4521.

32 A. Martini, S. A. Guda, A. A. Guda, G. Smolentsev, A. Algasov, O. Usoltsev, M. A. Soldatov, A. Bugaev, Yu. Rusalev, C. Lamberti and A. V Soldatov, *Comput. Phys. Commun.*, 2020, **250**, 107064.
